# Functional expression, characterization and application of the human S100A4 protein

**DOI:** 10.3892/mmr.2014.2745

**Published:** 2014-10-22

**Authors:** DEGANG WANG, JIANWEI ZHANG, ZIQUAN LIU, YUNYUN CHEN, CHUANXIANG XU, ZHIQING ZHANG, XIAOHUA LIU, LEI WU, XUESI ZHOU, XIANGYAN MENG, HUA LI, HONGTAO LIU, ZIFENG JIANG, TIANHUI WANG

**Affiliations:** 1 Performance Medicine Laboratory, Institute of Health and Environmental Medicine, Tianjin 300050, P.R. China; 2 Laboratory of Environmental Science, National Center of Biomedical Analysis, Haidian, Beijing 100850, P.R. China; 3 Department of Health and Exercise Sciences, Tian Jin University of Sport, Tianjin 300381, P.R. China; 4 Department of Physiology and Pathophysiology, Logistics College of Chinese People’s Armed Police Force, Tianjin 300162, P.R. China; 5 Endoscopy Division, Tianjin Medical University Cancer Hospital and City Key Laboratory of Tianjin Cancer Center, Tianjin 300060, P.R. China; 6 Division of Zoological Systematics and Evolution, Institute of Zoology, Chinese Academy of Sciences, Chaoyang, Beijing 100101, P.R. China

**Keywords:** human S100A4 protein, monoclonal antibody, preparation, application

## Abstract

Preparations utilizing monoclonal antibodies against S100A4 provide useful tools for functional studies to investigate the clinical applications of the human S100A4 protein. In the present study, human S100A4 protein was expressed in *Escherichia coli* (*E. coli*) BL21 (DE3), successfully purified by diethylaminoethyl cellulose anion-exchange chromatography and identified by western blot analysis. Soluble S100A4 bioactivity was confirmed by Transwell migration and invasion assays in the human HeLa cell line. Monoclonal antibodies (mAbs) were generated utilizing the standard hybridoma method and were validated by enzyme-linked immunosorbent assay and western blot analysis. The antibody was then used to examine human gastric carcinoma specimens by immunohistochemistry. Recombinant S100A4 was functionally expressed in *E. coli* and promoted the migration and invasion of HeLa cells. Four hybridoma cell lines, which secreted mAbs specifically against human S100A4 protein, were obtained. One of the four mAbs, namely 2A12D10B2, recognized human S100A4 as indicated by immunohistochemical staining of human gastric carcinoma specimens and recombinant S100A4 was functionally expressed in *E. coli*. The mAbs of recombinant S100A4 were suitable for detecting S100A4 expression in human tissues and for investigating the subsequent clinical applications of the protein.

## Introduction

S100A4 (metastasin, Mts1) belongs to a multifunctional S100 family of Ca^2+^-binding proteins with low molecular weight (9–13 kDa) that comprise >20 members (1,2). S100 proteins share the common feature of two Ca^2+^-binding EF-hand motives and do not possess enzymatic activity. S100 proteins regulate the activity of bound protein partners. Thus, each S100 dimer is capable of binding and facilitating the functional cross-bridging of two target proteins. The S100A4 is capable of forming homo-oligomers and heterodimers with S100A1, thereby increasing the functional potential of S100A4. To the best of our knowledge, S100A4, which is a polypeptide containing 101 amino acids with a molecular weight of 11.5 kDa, is associated with the Ca^2+^-dependent regulation of intra- and extracellular activities, including protein phosphorylation, enzyme activity and cell motility (3,4). Studies on the role of S100A4 have mainly focused on the invasive growth and metastasis of numerous types of cancer (5–7). Increasing S100A4 expression is closely correlated to breast, colorectal and gastric carcinomas. Several studies have indicated that S100A4, as a complementary specific index, may be used for early diagnosis and prognosis (8,9). Therefore, the possibility of generating whole S100A4 by recombinant techniques is greatly advantageous for such applications. However, studies on the biochemical roles and distribution of S100A4 protein and antibody have been hampered by technical limitations, including preparation difficulty and cross-reactivity of available antibodies.

The present study described the construction and expression of a synthetic gene that encodes S100A4 in *Escherichia coli* (*E. coli*). The bioactivity was also examined by Transwell migration and invasion assays. By utilizing soluble human S100A4 protein as an antigen, mouse models were vaccinated and four hybridoma cell lines were produced to generate antibodies against S100A4. One of the four monoclonal antibodies (mAbs) generated against S100A4, namely 2A12D10B2, was selected for further study due to its leading characteristics and functionality in western blot analysis, indirect ELISA and immunohistochemistry assay.

## Materials and methods

### Reagents

Expression vector pET32a (+), *E. coli* strain BL21 (DE3), HeLa (obtained from the China General Microbiological Culture Collection Center, CGMCC, Beijing, China) and SP2/0 (obtained from the Cell Resource Center, IBMS, CAMS/PUMC, Beijing, China) cell lines were preserved in our laboratory. Cells were not contaminated. The approximate passage number for the Hela and SP2/0 cell lines was 3 and 5, respectively). *E. coli* was cultured on a rotary shaker at 37°C. HeLa and SP2/0 cells were cultured in an RPMI-1640 medium supplemented with 10% fetal bovine serum (FBS) and 2 mM l-glutamate (all obtained from Gibco Life Technologies, Grand Island, NY, USA) at 37°C in a 5% CO_2_ humidified atmosphere. The gel extraction kit, plasmid extraction kit, Ndel, Xhol and T4 DNA ligase were purchased from Takara Bio, Inc. (Shiga, Japan). Acrylamide, *N*,*N*-methylene bisacrylamide, ion exchange chromatographic column and HiTrapTM protein G HP were obtained from GE Healthcare (Little Chalfont, UK). Matrigel was acquired from BD Biosciences (Franklin Lakes, NJ, USA). Transwell chambers were procured from EMD Millipore (Billerica, MA, USA). Complete and incomplete Freund’s adjuvants were obtained from Sigma-Aldrich (St. Louis, MO, USA). β-Tubulin (H-235) was purchased from Santa Cruz Biotechnology, Inc. (Santa Cruz, CA, USA). Horseradish peroxidase (HRP)-conjugated goat anti-mouse immunoglobulin (Ig)G secondary antibody was procured from Beijing ZhongShan Golden Bridge Biotechnology Co., Ltd., (Guangdong, China). All other chemicals utilized were of analytical grade and obtained from Shanghai Sangon Biological Engineering Technology and Service Co., Ltd., (Shanghai, China). Ultra-pure water was used in this study. Other reagents and materials were purchased from Sigma-Aldrich (St. Louis, MO, USA). This study was approved by the committee of the Institute of Health and Environmental Medicine, Tianjin, China. The gastric tissues samples were obtained from the Tianjin Medical University Cancer Hospital and City Key Laboratory of Tianjin Cancer Center. The animals were obtained from the Experimental Animal Room of the Institute of Health and Environmental Medicine (Tianjin, China).

### Construction of expression vector pET32a-S100A4

A gene fragment of human S100A4 was constructed by overlapping polymerase chain reaction (PCR) with eight synthetic oligonucleotides as primers (Table I). The primers were synthesized based on the codon preference in *E. coli*. The forward primer (5′-GCCATATGGCGTGCCCGCTGGAAAAAG-3′) contained the Ndel restriction site (underlined), whereas the reverse primer (5′GCGCTCGAGTCATTTTTTGCGCGGCTGTTTATCCG-3′) contained the Xhol restriction site (underlined). The expression vector pET32a-S100A4 was then constructed utilizing the standard method and the nucleotide sequence of the target gene was confirmed in the positive clones by DNA sequencing.

### Expression, purification and identification of recombinant S100A4

Recombinant plasmids pET32a-S100A4 were transferred into *E. coli* BL21 (DE3) and induced to express the S100A4 protein by isopropyl-β-d-thiogalactopyranoside (IPTG). Following this, the cell lysate was sonicated and centrifuged. Protein samples from the supernatant, precipitation and cell lysate were analyzed by SDS-PAGE and stained with Coomassie brilliant blue to confirm the pET32a-S100A4 expression. The supernatant sample was purified by ion exchange chromatography column (HiTrap™ DEAE FF; loading buffer: Tris-HCl 0.02mol/l, pH 8.8; and elution buffer: 50mM/100mM/200mM/500mM NaCl Tris-HCl 0.02mol/l, pH 8.8; GE Healthcare Life Sciences, Chalfont, UK) according to the instructions of the manufacturer. Protein concentration was measured with a Bradford protein assay kit (Boster, Wuhan, China) using bovine serum albumin (BSA; Huasheng Biotech, Inc., Tianjin, China) as a standard (10).

### Bioactivity of recombinant human protein S100A4

To determine whether the recombinant S100A4 protein stimulated cell migration and invasion, cell motility and invasion assays were conducted in a Transwell chamber (EMD Millipore) (11), which was coated with Matrigel^®^. Assays were conducted according to the manufacturer’s instructions. Following overnight starvation in RPMI-1640 media and the addition of a medium containing 10% FBS to the bottom chambers as a chemoattractant, HeLa cells (1×10^5^) were added to the top chambers of the 24-well transwell plates as the control group (C), whereas HeLa cells (1×10^5^) with recombinant human S100A4 were added into another top chamber as the experimental group (E). The cells were then incubated for 24 h at 37°C. Non-motile cells on top of each chamber were removed by wiping with cotton swabs. The cells on the bottom of each chamber were fixed with 0.1% glutaraldehyde for 30 min, rinsed briefly with phosphate-buffered saline (PBS) and stained with 0.2% crystal violet (Ameresco, Inc., Framingham, MA, USA) for 20 min. The chambers were then washed thoroughly with PBS. The number of migrated or invaded cells was calculated under ×200 magnification and the mean for each chamber was determined. The results were calculated as the migration/invasion rate as compared with the parental control cells. Each experimental condition was conducted in duplicate and repeated at least three times.

### Preparation of mAbs against human S100A4

The procedures for mAb preparation were performed as previously described (12). Female BALB/c mice (obtained from the Experimental Animal Room of Institute of Environmental Medicine Research; weight, 18–30g; age, 8–10 weeks). at 8–10 weeks of age were immunized three times over the course of two weeks with purified recombinant S100A4 protein. Mice were housed up to four per cage and maintained at 22–25°C, under an alternating 12-h light/dark cycle. Mice were placed on a standard diet and allowed access to food and water, ad libitum. The mice were then sacrificed by cervical dislocation and the splenocytes were collected and fused with SP2/0 myeloma cells utilizing the PEG method. Hybridoma cells were selected in the hypoxanthine, aminopterin and thymidine media (media obtained from Invitrogen Life Technologies, Carlsbad, CA, USA and supplements from Sigma-Aldrich). Positive clones were confirmed by indirect ELISA and positive hybridoma cell lines were obtained following three subcloning cycles. To generate anti-S100A4 mAbs, hybridoma cells were injected intraperitoneally into liquid paraffin-primed female BALB/c mice (8–10 weeks old) at ~1×10^6^ cells/mouse. Following 1 week, ascites were collected and antibodies were purified by protein G affinity chromatography. According to the manufacturer, antibodies were purified by Histrap protein G HP (1 ml),(GE Healthcare Life Sciences). The loading buffer was 20 mM PB, pH 7.0 and the elution buffer was 0.1 mol/l Gly-HCl, pH 2.7.

### ELISA

Antibody titers were determined by indirect ELISA as described previously (13). ~100 μl of recombinant S100A4 (4 μg/ml) was added into the microtiter plates. The plates were then incubated overnight at 4°C. Following washing three times with PBS containing 0.05% Tween-20 (PBST), the plates were blocked with 3% BSA in PBS containing Tween 20 for 1 h at 37°C. Different serum dilutions from immunized mice, cell culture supernatants or ascites were added to the plates for 2 h at 37°C. The plates were then incubated with HRP-coupled goat anti-mouse IgG for 1 h and with tetramethyl benzidine as substrate for 10 min. The colorimetric signal was measured at A450 and A630 nm. The specification and affinity constants of the mAbs were determined by competitive ELISA as described previously (14,15) (Table II).

### SDS-PAGE and western blot analysis of recombinant S100A4

Bacterial extracts or lysates of the cells were separated by SDS-PAGE under reducing or non-reducing conditions on a 12% polyacrylamide gel, and then transferred onto polyvinylidene fluoride membranes (Millipore) by electroblotting in vertical buffer tanks. The membranes were blocked with 5% non-fat milk in Tris-buffered saline/Tween-20 buffer (50 mM Tris+HCl, pH 7.4, 0.9% NaCl and 0.1% Tween-20; CWBIO, Beijing, China) and incubated with rabbit polyclonal antibody against human S100A4 for 2 h at room temperature or overnight at 4°C. Following the addition of the HRP-coupled goat anti-rabbit IgG for 45 min, the membrane bands were detected by the enhanced chemiluminescent system (Pierce Biotechnology, Inc., Rockford, IL, USA).

### Cross-reaction

Recombinant S100A4 and its analogues were used for the cross-reactivity (CR) study in the competitive ELISA. Recombinant S100A4 antigen, dissolved in PBS was coated in the ELISA plate, and serial diluted proteins (i.e., recombinant S100A4, S100A1 and S100B) were pre-incubated with S100A4 mAbs for 30 min. The mixture was then incubated with the coated antigen. CR rate from the proteins was expressed as a ratio of the analyte concentration to the half-maximal inhibitory concentration (IC_50_; ng/ml). The IC_50_ and CR values were calculated according to the formulas demonstrated in equations (1) and (2), respectively.

(1) $$IC\;(\%)=\frac{A-A_{ex}}{A_{0}-A_{ex}}\times 100$$

where A is the absorbance of the protein under standard concentration, A_0_ is the absorbance under zero protein concentration and A_ex_ is the absorbance under excessive protein concentration.

(2) $$CR\;(\%)=\frac{IC_{50}\;of\;S100A4}{IC_{50}\;of\;S100A4\;anal\text{ogue}}\times 100$$

### Affinity analysis

mAb affinity against S100A4 was assessed by surface plasmon resonance (SPR; AutoLab, Utrecht, Netherlands). S100A4 was immobilized on a carboxylated sensor chip (Metrohm Auto Lab) with standard amine coupling in sodium acetate buffer (pH 4.5) according to the manufacturer’s instructions. Extracted mAbs were placed onto the immobilized chip surface. The results were analyzed by Autolab ESPRIT Data Acquisition 4.3 and Kinetic Evaluation 5.0 software (Metrohm Auto Lab). The affinity of the selected mAbs is expressed as dissociation constants.

### Immunohistochemistry

Paraffin-embedded human gastric cancer tissues were obtained from ten patients who had undergone surgery and had a confirmed diagnosis of gastric cancer, at Tianjin Medical University Cancer Institute and Hospital (Tianjin, China). One sample was obtained from each patient. Written informed consent was obtained from the patients. Tissue sections were prepared into 5-μm thick slices. The sections were deparaffinized in xylene and ethanol and then rehydrated in distilled water. The sections were submerged in EDTA antigenic retrieval buffer and microwaved for antigen retrieval. The sections were then treated with 3% hydrogen peroxide in methanol to quench endogenous peroxidase activity followed by incubation with 1% BSA to block non-specific binding. The sections were then incubated with the primary antibody, which had been produced as aforementioned, and mouse anti-S100A4 (R&D Systems, Inc., Wiesbaden, Germany) at 4°C overnight. S100A4 was stained in a buffy color in the cytoplasm and nucleus. The immunostaining degree was reviewed and scored independently by two observers based on staining intensity (16,17). Staining intensity was graded according to the following criteria (18): Zero (no staining), one (weak staining, light yellow), two (moderate staining, yellow brown) and three (strong staining, brown). Moderate and strong staining was considered to indicate high S100A4 expression, whereas no and weak staining was considered to indicate low S100A4 expression in tumors.

## Results

### Expression and purification of human recombinant protein S100A4

**Figure 1:**
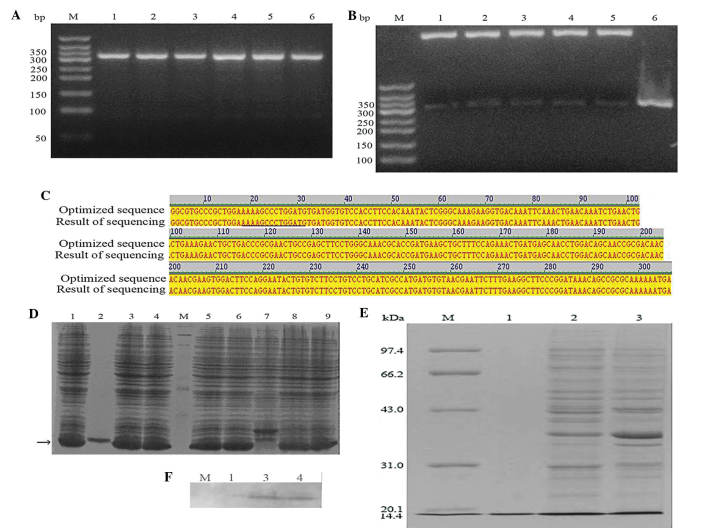
Recombinant expression and purification of human S100A4 protein. (A) Agarose gel analysis of human S100A4 cDNA. Lanes 1–6, human S100A4 cDNA; lane M, DNA marker. (B) Agarose gel analysis of the pET32a-S100A4 vector following restriction enzyme treatment by using Ndel and Xhol. Lanes 1–5, pET32a-S100A4 digested by Ndel and Xhol; lane 6, positive control; lane M, DNA marker. (C) DNA sequencing result. (D) Recombinant expression of pET32a-S100A4. Lane 2, positive control; lane M, protein standards; lanes 1, 3–6, 8–9, induced products of pET32a-S100A4; lane 7, negative control. (E) SDS-PAGE analysis of bacterial cultures in the sediment fraction or supernatant fraction. Lane 1, positive control; lane M, protein standards; lane 2, induced products of pET32a-S100A4 in the supernatant fraction; lane 3, induced products of pET32a-S100A4 in the sediment fraction. (F) Western blot analysis of the recombinant protein. Lane M, protein standards; lane 1, negative control; lane 2, induced products of pET32a-S100A4 in the supernatant fraction; lane 3, induced products of pET32a-S100A4 in the sediment fraction.

The gene fragment of human S100A4 was assembled by PCR. The full length of the gene fragment was 306 bp (Fig. 1A). The enzyme digestion identification and DNA sequencing confirmed that the human S100A4 gene was successfully cloned into the pET32a in the correct orientation (Figs. 1B and C). The recombinant plasmid pET32a-S100A4 was transformed to an expression host, namely, *E. coli* BL21 (DE3). The recombinant protein had a molecular weight of approximately 11.5 kDa, which is consistent with the estimated size (Fig. 1D; lanes 1, 3–6, 8–9). However, this band was not observed in the negative control samples (Fig. 1D; lane 7). The expressed recombinant protein was identified in the supernatant and in an insoluble form as inclusion bodies (Fig. 1E). To characterize the recombinant protein further, western blotting was performed using an goat anti-human S100A4 antibody. The recombinant protein reacted with the antibody, thus indicating the successful expression of recombinant human S100A4 protein in *E. coli* BL21 (DE3) cells (Fig. 1F). The presence of the recombinant protein confirmed that the recombinant human S100A4 protein was successfully purified by using ion exchange chromatography.

### S100A4 protein increases migration and invasion of HeLa cells

**Figure 2:**
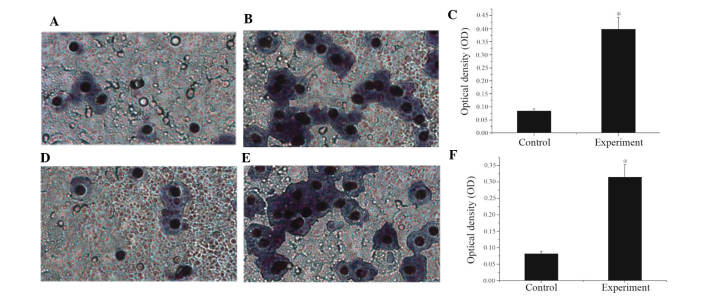
Increased invasion and migration of HeLa cells by the recombinant protein. (A) Transwell migration of HeLa cells (1×10^5^) transfected with pET32a plasmid. (B) Transwell migration of HeLa cells (1×10^5^) transfected with pET32a-S100A4 plasmid. (C) OD value of HeLa cells in the migration experiment group compared with the control group. (D) Transwell invasion of HeLa cells (1×10^5^) transfected with pET32a plasmid. (E) Transwell invasion of HeLa cells (1×10^5^) transfected with pET32a-S100A4 plasmid. (F) OD value of HeLa cells in the invasion experiment group compared with the control group. OD, optical density. *P<0.05, compared with control.

Transwell migration and invasion assays demonstrated an increased number of cells in the experimental group compared with that in the control group (Fig. 2A–D), thus indicating that the recombinant S100A4 protein promoted the motility and invasiveness of cancer cells. A four-fold increase was observed in the number of migrating cells in the experimental group (0.36±0.05) compared with that in the control group (0.09±0.01). The invasive ability of HeLa cells in the experimental group (0.24±0.04) was 300% greater than that of the control cells (0.08±0.01) as confirmed by the Matrigel invasion assay. This result suggested that recombinant human S100A4 protein promoted the motility and invasiveness of cancer cells.

### Preparation and characterization of anti-S100A4 mAbs

**Figure 3:**
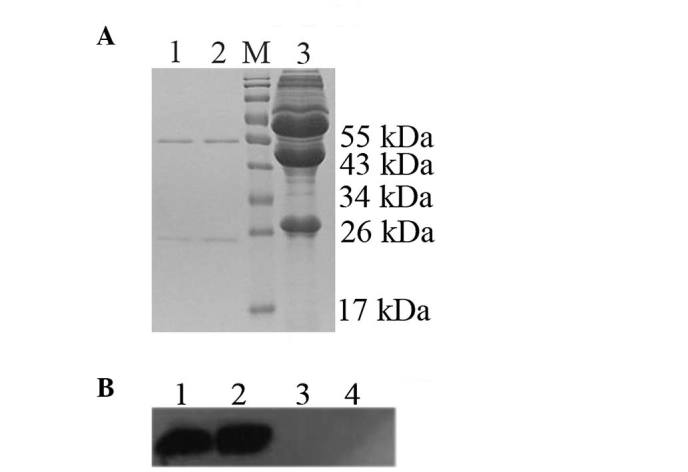
SDS-PAGE and western blot analysis of purified S100A4 mAbs. (A) Proteins were separated by 12% SDS-PAGE and stained with Coomassie brilliant blue. Lanes 1 and 2, purified ascitic fluid; lane 3, unpurified ascitic fluid; lane M, molecular size markers. (B) Western blot analysis of purified S100A4 mAbs with antigen. Lanes 1 and 2, purified ascites of BALB/c mice injected hybridoma cell lines 2A12D10B2 and 3A5G8C6; lanes 3 and 4, purified ascites of BALB/c mice injected with SP2/0 myeloma cells.

To study the function of S100A4 further, murine mAbs against S100A4 were prepared. Four hybridoma cell lines, namely 2A12D10B2, 3A5G8C6, 3G9C5B6 and 3G9C5F8, were obtained following immunization of mice with soluble S100A4 as the antigen, cell fusion and hybridoma cell cloning and subcloning. These cell lines stably produced anti-S100A4 mAbs (Table II). Highly concentrated S100A4 mAbs were prepared from BALB/c mouse ascites and purified by protein G affinity chromatography. The purity and molecular weight of S100A4 mAbs was determined by SDS-PAGE analysis (Fig. 3A). The specificity of the S100A4 mAbs were determined by western blot analysis (Fig. 3B).

The CR, along with proteins that have similar structures and functions to human S100A4, is an important parameter for evaluating the specificity of an immunoassay. The results summarized in Table III revealed that 2A12D10B2 had 100% CR to human S100A4 and 3.14%, 1.29% and 0.047% CRs to mouse S100A4, human S100A1 and S100B, respectively (Table III).

**Figure 4:**
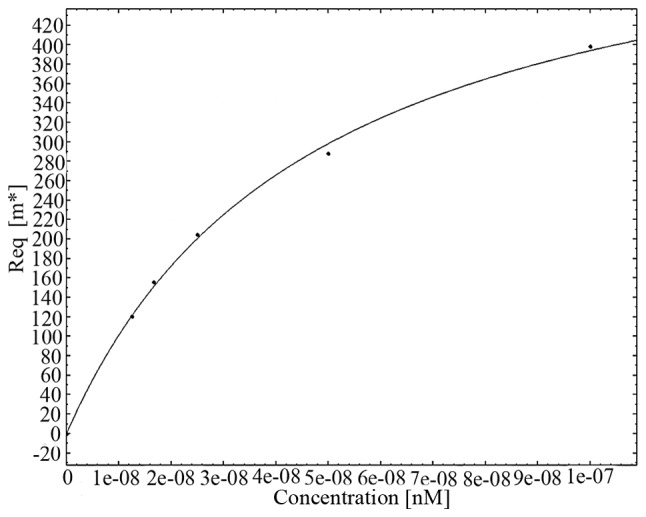
Plotted standard curve based on SPR data. SPR, surface plasmon resonance; Req, response of equilibrium.

To calculate the association rate constant, 2A12D10B2 mAb was appropriately diluted in PBS and analyzed by SPR at several concentrations (Fig. 4). The equilibrium dissociation constant (KD) for the 2A12D10B2 clone was also calculated. KDs were determined independently by Kinetic Evaluation 5.0 software. The KD of 2A12D10B2 was ~4.72×10^−8^ mol/l.

### Application of antibodies in immunohistochemistry assay

**Figure 5:**
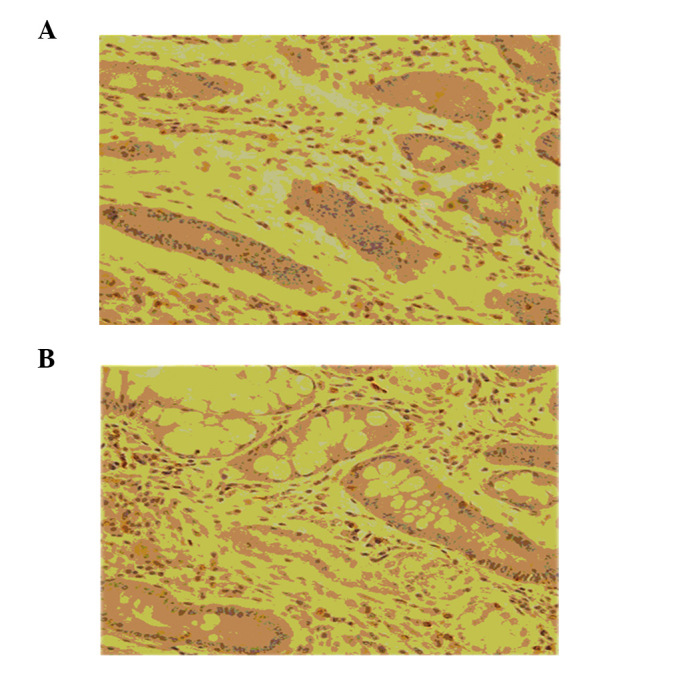
Immunohistochemical staining of human gastric carcinomas with 2A12D10B2 and commercial mouse anti-S100A4 mAb. (A) Immunostaining of gastric carcinomas with 2A12D10B2 (magnification, ×400). (B) Immunostaining of gastric carcinomas with commercial mouse anti-S100A4 mAb (magnification, ×400). mAbs, monoclonal antibody.

One of the four hybridoma cell lines, namely 2A12D10B2, was further selected for subsequent tests due to its high growth characteristics *in vitro* and high S100A4 reactivity in the immunohistochemical assay. Immunoreaction products were detected in gastric carcinoma specimens and were mainly localized in the cell cytoplasm (Fig. 5). Immunohistochemical analysis revealed that the mAbs were suitable for detecting S100A4 expression in human gastric carcinoma specimens.

## Discussion

The S100A4-encoding protein is a member of the S100 gene family and is a polypeptide containing 101 amino acids with a molecular mass of ~11.5 kDa (6) Growing evidence indicates that the S100A4 protein, which exists as a non-covalent dimer within cells, may be secreted into the extracellular space and form covalently linked dimers in the intercellular substance. Therefore, the S100A4-encoding protein reflects the structural plasticity of the S100A4 protein and provides a structural basis for biological function diversity *in vivo* (7).

Increasing evidence suggested that S100A4 protein modulates numerous cell functions, including cell growth, intracellular signal transduction, cell contractility and cell-cell communication (8–10). Previous studies on S100A4 protein have focused on its potential use in the field of oncology. Recent studies have demonstrated that S100A4 protein is closely associated with tumor growth, progression and patient outcome (11). Therefore, the S100A4 protein is an important factor for promoting metastasis. In humans, S100A4 is not detected in normal tissues obtained from the lung, kidney, breast, thyroid, pancreas and colon (12–14). By contrast, high S100A4 expression has been observed in breast cancer, colorectal tumors, gastric cancer, pancreatic carcinoma, esophageal squamous cell carcinoma and osteosarcoma cells (15–17). S100A4 protein is closely associated with tumor invasiveness and metastasis. In addition to the elucidation of the molecular mechanisms underlying the role of S100A4 in cancer, the S100A4 protein may have clinical utility as a prognostic, predictive or diagnostic tool and may provide a novel method for the prevention and treatment of tumor metastasis.

In the present study, the human S100A4 sequence was optimized and synthesized according to the codon usage bias of *E. coli*. The expression vector pET32a-S100A4 was then constructed by linking the S100A4 sequence with the pET32a plasmid. Following confirmation by DNA sequencing, the recombinant expression plasmid (i.e., pET32a-S100A4) was transferred into competent *E. coli* BL21 (DE3) cells. Human recombinant S100A4 protein was functionally expressed in *E. coli* BL21 (DE3) at a high level, ~20% of the total protein content in the bacterial lysates induced by IPTG. Western blot analysis suggested that the protein expression was targeted by S100A4 protein. High-purity S100A4 protein (95% by SDS-PAGE) may be used for basic investigations into the interaction with p53 or other target molecules.

Several methods are used to verify the activity of recombinant proteins (16,18). The present study designed the Transwell migration and invasion assays to verify the bioactivity of the recombinant S100A4 protein. The data demonstrated that recombinant protein levels affect the motility and invasiveness of cancer cells.

In conclusion, in the present study, the human S100A4 protein was successfully expressed in *E. coli* and an efficient method was developed for producing biologically active S100A4. By using soluble S100A4 as an antigen, mAbs were prepared against human S100A4. One of the mAbs, namely 2A12D10B2, was functional in several applications, including western blotting, immunohistochemistry assay and ELISA. Therefore, soluble human S100A4 protein and its mAbs may be used as tools for further functional investigations into S100A4 and for the improvement of diagnostic and therapeutic strategies in cancer treatment.

## Figures and Tables

**Table I:** Oligonucleotide primers with mutual overlaps.

Primer	Primer sequence	Primer length (bp)
1	ATGGCGTGCCCGCTGGAAAAAGCCCTGGATGTGATGGTGTCCACCTTCCACAAAT	55
2	GTTCAGTTTGAATTTGTCACCTTCTTTGCCCGAGTATTTGTGGAAGGTGGACACC	55
3	ACAAATTCAAACTGAACAAATCTGAACTGAAAGAACTGCTGACCCGCGAACT	52
4	GCAGCTTCATCGGTGCGTTTGCCCAGGAAGCTCGGCAGTTCGCGGGTCAGCAGTT	55
5	CGCACCGATGAAGCTGCTTTCCAGAAACTGATGAGCAACCTGGACAGCAACCGCG	55
6	GGAAGACACAGTATTCCTGGAAGTCCACTTCGTTGTCGCGGTTGCTGTCCAGGTT	55
7	GGAATACTGTGTCTTCCTGTCCTGCATCGCCATGATGTGTAACGAATTCTTTG	53
8	TCATTTTTTGCGCGGCTGTTTATCCGGGAAGCCTTCAAAGAATTCGTTACAC	52
10432F	GCCATATGGCGTGCCCGCTGGAAAAAG	27
104TRXR	GCGCTCGAGTCATTTTTTGCGCGGCTGTTTATCCG	35

**Table II:** Features of S100A4 mAbs.

	3G9C5B6	3G9C5F8	2A12D10B2	3A5G8C6
Titer (ascitic fluid)	1:51,200	1:51,200	1:204,800	1:204,800
Affinity constant (l/mol)	2.62×10^8^	1.28×10^8^	5.79×10^8^	6.37×10^8^
Concentration (mg/ml)	6.15	4.62	34.2	6.8

Four hybridoma cell lines, namely 2A12D10B2, 3A5G8C6, 3G9C5B6 and 3G9C5F8, were obtained following immunization of mice with soluble S100A4 as an antigen, cell fusion and hybridoma cell cloning and subcloning. These cell lines stably produced anti-S100A4 mAbs. mAbs, monoclonal antibodies.

**Table III:** CRs of selected proteins.

	CR	
	
Protein	IC_50_ (ng/ml)	CR%
Human S100A4	3.25	100.00
Mouse S100A4	103.42	3.14
Human S100A1	251.32	1.29
Human S100B	682.84	0.047
Negative control	ND	ND

CR, cross-reactivity; IC_50_, half-maximal inhibitory concentration; ND, no detection.
